# Laser treatment of acne scars: a bibliometric analysis and descriptive summary of clinical evidence directions over the past 20 years

**DOI:** 10.3389/fmed.2026.1875002

**Published:** 2026-07-22

**Authors:** Chunping Ji, Chenxing Xie, Yinzi Qiao

**Affiliations:** 1 First Affiliated Hospital of Hebei North University, Hebei North University, Zhangjiakou, China; 2 Dongzhimen Hospital, Beijing University of Chinese Medicine, Beijing, China; 3 Beijing University of Chinese Medicine, Beijing, China

**Keywords:** acne scar, bibliometrics, combination therapy, cosmetic dermatology, laser, safety

## Abstract

**Objective:**

Laser therapy is widely used in the management of acne scars; however, a clear characterization of research hotspots and clinical evidence directions is lacking. The aim of the study was to examine the research status and emerging trends in laser therapy for acne scars and to descriptively summarize the clinical evidence directions over the period 2006–2026, providing a reference for future investigations.

**Methods:**

The Web of Science Core Collection was searched for relevant records published from January 1, 2006, to March 10, 2026. WoSCC records were analyzed using R software, VOSviewer, CiteSpace, and Origin. Additionally, PubMed was searched separately for meta-analyses during the same period to provide a supplementary descriptive summary of reported clinical evidence directions.

**Results:**

In total, 898 WoSCC records were included. Publications showed a sustained upward trend, particularly from 2010 to 2025. Records for 2026 were collected only up to March 10 and were therefore not included in the full-year trend interpretation. The United States led in output, with China, South Korea, Egypt, and Thailand following. Among the top ten countries by publication output, Germany showed a relatively high proportion of multi-country publications based on the combined interpretation of MCP% and total publication output. Mahidol University and Cairo University were representative cooperation centers. *Dermatologic Surgery* had the highest number of publications and citations. Ablative fractional lasers are commonly used based on the principle of selective photothermolysis, with published controlled trials reporting favorable efficacy outcomes. Combination therapies, including platelet-rich plasma, fillers, or isotretinoin, have emerged as prominent research focuses. Adverse events, including postinflammatory hyperpigmentation, have drawn increasing research attention to safety and individualized treatment strategies. Radiofrequency-based treatments have also attracted research interest because of their potential applicability across different skin types.

**Conclusion:**

Current studies increasingly focus on more refined laser approaches and their combination with other treatments, while research on personalized treatment strategies may represent an important direction for future research.

## 1 Introduction

Acne scars are a common lifelong sequela in patients with acne, resulting from inflammatory responses, infection, improper treatment, and other factors. These pathogenic processes damage the dermis and subcutaneous tissue, leading to the breakdown, loss, or aberrant proliferation of collagen and elastic fibers. Acne scars are clinically categorized into atrophic scars, hypertrophic scars, and keloids according to their pathological features. A meta-analysis by Tan et al. (2017) reported that approximately 43% of patients with acne develop scars of varying severity, with the incidence positively correlated with acne severity (1). Acne scars are often difficult to treat and may significantly affect patients’ physical and mental health. Early intervention and anti-inflammatory repair are therefore essential to prevent further skin damage and the formation of prominent scars. Additionally, controlling excessive sebaceous secretion and reducing inflammatory injury are effective strategies to decrease the risk of acne scarring. Current dermatological treatment strategies for acne scars primarily involve external interventions, including phototherapy, surgery, filler injections, and chemical exfoliation (2). These approaches are often combined with clinical drug therapy to enhance efficacy. Topical medications, such as silicone preparations and heparin sodium allantoin, and local injections, including glucocorticoids, botulinum toxin type A, fluorouracil, and bleomycin, are commonly employed (3, 4). However, acne scars are frequently irreversible, with the highest risk occurring during youth. Scarring can lead to negative psychological outcomes, maladaptive coping behaviors, and a reduced quality of life (5). Current clinical treatments often have limitations: monotherapy rarely achieves ideal outcomes and may be associated with adverse effects (6). Moreover, some treatments are effective only for specific types of acne scars—for example, photodynamic therapy is generally limited to patients with *Propionibacterium acnes*-dependent moderate to severe acne (7). Consequently, there is a critical need to develop new, effective treatment strategies to improve the quality of life for patients with acne scars.

Laser cosmetology is a medical aesthetic technology that utilizes lasers of specific wavelengths to target skin tissues. Through the principle of selective photothermal action, lasers can selectively decompose pigment cells without causing significant damage to surrounding healthy tissues, thereby achieving therapeutic effects for acne, freckles, wrinkles, and other skin concerns. The application of lasers in dermatology began in 1963 when Goldman successfully used a ruby laser to treat benign skin lesions and tattoos (8), establishing a precedent for the medical use of lasers. With technological advancements, systematic treatment protocols have been developed for acne scars. To inhibit sebaceous gland secretion and reduce inflammation, various near-infrared wavelength lasers—such as the 1,320 nm, 1,450 nm, and 1,550 nm lasers—are commonly employed (9). For the treatment of post-inflammatory erythema, intense pulsed light and pulsed dye lasers are frequently used (10). Non-ablative fractional lasers (1,440 nm, 1,540 nm, and 1,550 nm) and ablative fractional lasers (2,940 nm, 10,600 nm) are widely applied to improve acne scarring (11). Clinically, small spot sizes, low energy levels, and low lattice density parameters (12), are often employed in multiple treatment sessions to optimize efficacy while ensuring patient safety. Despite the early adoption of laser therapy for acne scars, standardized treatment guidelines and long-term safety data remain limited, particularly across different scar types, stages, and skin types (13). Unified risk stratification standards and long-term follow-up studies are lacking (14), and controversies persist regarding both safety and long-term efficacy. Studies have reported that prolonged or high-energy laser treatments, particularly with near-infrared and ablative fractional lasers, may have lasting effects on skin barrier function, dermal collagen metabolism, and local microcirculation, potentially leading to irreversible skin sensitivity, persistent erythema, or occult scar formation (15). Although extensive research has been conducted regarding laser therapy for acne scars, the absence of clear overviews of key research trends and clinical evidence directions within this domain hampers researchers’ ability to quickly and accurately understand current trends and identify future research directions.

As an important tool for the quantitative analysis of disciplinary development, bibliometrics can reveal the evolution, research hotspots, and frontiers of a specific field through data mining and visualization techniques. In the present study, a WoSCC-based bibliometric analysis of laser therapy for acne scars was performed using software tools such as VOSviewer, R, and CiteSpace to examine research published between 2006 and 2026 and visualize research hotspots. Separately, the clinical evidence directions reported in PubMed-indexed meta-analyses were summarized. This study provides scholars with insights into key knowledge frontiers and a reference for future research on treatment strategies.

## 2 Materials and methods

### 2.1 Data sources and retrieval strategies

Data were collected on March 10, 2026, through separate searches of the Web of Science Core Collection (WoSCC) and the PubMed database. In the WoSCC database, the following search strategy was applied: ((((TS = (Acne Scar*))AND TS = (Laser*))AND DOP = (2006-01-01/2026-03-10))AND DT = (Article OR Review)) AND LA = (English). The WoSCC dataset therefore comprised both Articles and Reviews, and all WoSCC-based bibliometric analyses were performed using this combined dataset. In the PubMed database, the search strategy was as follows: ((Acne Scar*[Title/Abstract]) AND (Laser*[Title/Abstract])) AND ((“2006/01/01”[Date - Completion]: “2026/03/10”[Date - Completion])) Filters: Meta-Analysis, English. The retrieval process is illustrated in Figure S1. Records retrieved from WoSCC were exported in plain text format as complete records, including cited references. Records retrieved from PubMed were exported in PubMed format. To define the scope of the PubMed dataset, the inclusion criteria for the full-text eligibility assessment were as follows: (1) studies evaluating laser-based treatments for acne scars; (2) laser types including fractional lasers (ablative or non-ablative), pulsed dye laser (PDL), laser-assisted photodynamic therapy (PDT), near-infrared lasers, KTP lasers, and other medical-grade laser or energy-based devices; and (3) studies involving medical-grade laser or energy-based treatment devices. The exclusion criteria were as follows: (1) animal or *in vitro* studies; (2) studies mainly evaluating non-laser light-based therapies, such as blue light, red light, or ultraviolet light without a laser component; and (3) studies of home-use low-energy laser/light devices, namely non-medical-grade devices. During full-text eligibility assessment, 27 PubMed Meta-Analysis articles were assessed. Two publications were excluded because they did not meet the predefined criteria: one primarily evaluated microneedling and its combinations rather than laser- or medical-grade energy-based treatment, and the other focused on multiple scar types rather than laser treatment for acne scars. Finally, 25 PubMed Meta-Analysis articles were included.

### 2.2 Data analysis

Due to differences in data formats between the two databases, separate analyses were conducted for each to minimize data loss and ensure more rigorous results. WoSCC data contain relatively complete citation information and standardized bibliometric fields, making them more suitable for bibliometric mapping analyses such as annual publication trends, country/institutional collaboration, journal co-citation, keyword co-occurrence, and trend evolution. Therefore, WoSCC was used as the main source for bibliometric analysis to describe the overall knowledge structure, research themes, and hotspots of this field over the past 20 years. Given the large number of clinical studies in this field, PubMed Meta-Analysis articles were additionally retrieved to describe the main reported clinical evidence directions. The 25 finally included meta-analyses were used as supplementary descriptive evidence to present the main clinical research concerns and evidence distribution. The PubMed Meta-Analysis section was not used for keyword co-occurrence or trend mapping analysis and was not merged with the WoSCC dataset. Following established bibliometric methods, trends in annual publications were assessed using Origin 2018. Additionally, visualization and analysis were performed using R software (v4.5.2) with the bibliometrix package (v5.2.1), VOSviewer (v1.6.20.0), and CiteSpace (v6.4. R1). Bibliometrix is an open-source tool for visual analysis and mapping. Collaboration between countries and institutions, along with journal citation-based associations and keyword co-occurrence patterns, were visualized using VOSviewer. Specific parameters used in VOSviewer analyses are as follows: (1) The national co-authorship network included countries with at least 5 publications. In the interpretation of the proportion of multi-country publications (MCP%), MCP% was described only among the top ten countries by publication output and interpreted together with each country’s total publication output to reduce potential bias caused by small absolute publication numbers. (2) The institutional co-authorship network included institutions with at least 4 publications. (3) Source co-citation analysis considered sources with a minimum of 30 citations. (4) Keyword co-occurrence analysis included keywords appearing at least 7 times. To reduce the influence of semantic duplication and differences in keyword expression, keywords were standardized before analysis. Synonymous keywords were merged with reference to Medical Subject Headings (MeSH) and Chinese Medical Subject Headings (CMeSH) controlled vocabularies. The merging process was independently conducted by two researchers and cross-checked; disagreements were resolved through discussion. The complete list of merged synonym terms is provided in Supplementary material 1. The 25 PubMed meta-analyses retained in the final dataset were summarized as supplementary descriptive evidence and were not independently reanalyzed to reassess clinical efficacy. We extracted the title, main content/intervention, outcomes/effect measures, main reported results, and quality assessment or risk-of-bias information when reported in the source publications or available abstracts. Effect-measure information included OR, RR, MD, WMD, SMD, SUCRA, or *p* values when available, depending on the reporting format of the original meta-analysis. The relevant information is summarized in Supplementary material 2. Because the included meta-analyses differed in study types, interventions, outcome indicators, and effect-measure reporting, this study did not perform a new pooled analysis of the PubMed meta-analysis results or an independent AMSTAR 2 quality rating. Effect-measure information and quality/risk-of-bias information were extracted only as reported in the source meta-analyses; the main text presents these data descriptively, and the clinical implications and research insights are interpreted cautiously in the Discussion. The journal impact factors were obtained based on 2024 data from the Journal Citation Reports.

## 3 Results

### 3.1 Research landscape of laser treatment for acne scars

**Figure 1:**
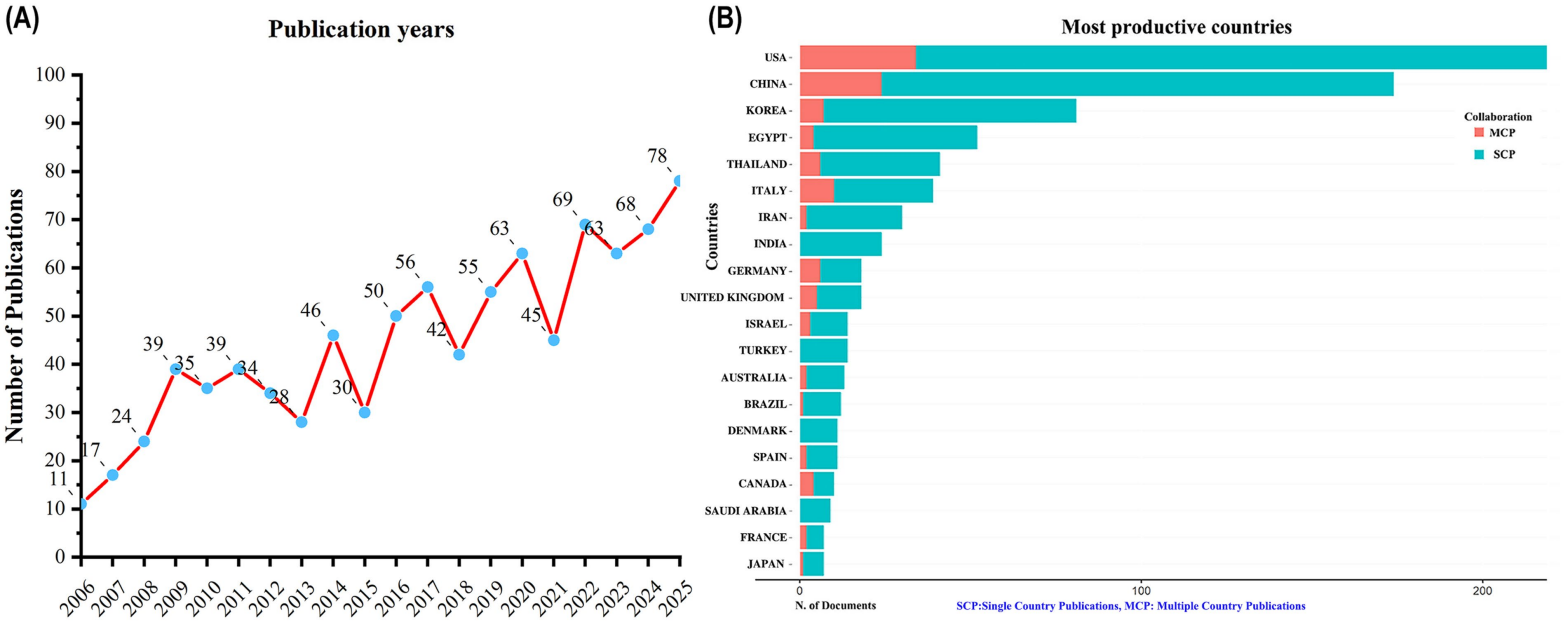
Trends in annual publication outputs in the field of laser treatment for acne scars. **(A)** Trends of annual publication outputs based on complete calendar years from 2006 to 2025. **(B)** Distribution of corresponding authors’ countries and cooperation based on records retrieved from 2006 to March 10, 2026.

In total, 898 unique WoSCC records were identified, including 705 Articles and 193 Reviews, and subsequent WoSCC-based bibliometric mapping analyses were performed using this combined dataset. As illustrated in Figure 1A, publication output in this field exhibited a strong and sustained growth from 2006 to 2025, with a steady increase observed between 2010 and 2025 despite minor fluctuations. The year with the highest number of publications was 2025, with a total of 78 articles. Because the 2026 data were collected only up to March 10, they are presented only as partial retrieval results and were not included in full-year trend comparisons.

**Figure 2:**
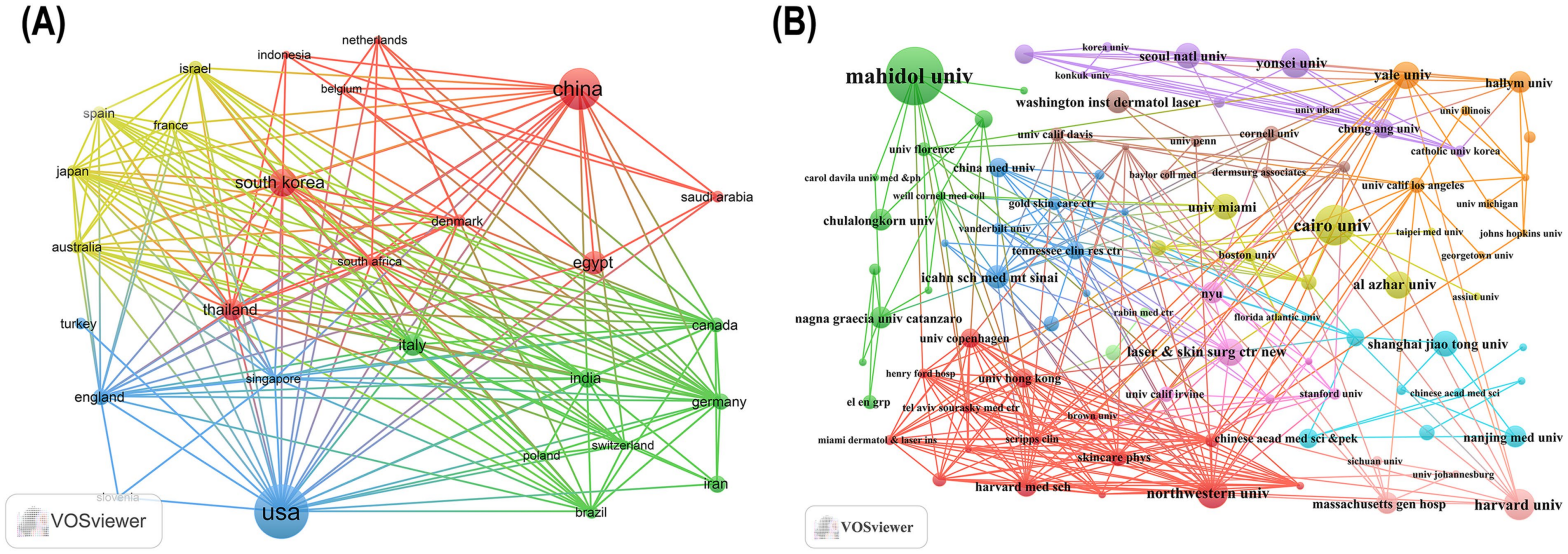
Map of countries/regions and institutions involved in the research on laser treatment for acne scars, 2006–2026. **(A)** Map of cooperation between different countries. **(B)** Map of cooperation between different institutions.

The five most productive countries in this field included the United States (260), China (174), South Korea (81), Egypt (52), and Thailand (41), with the United States ranking first in overall research output. Among the top ten countries by publication output, Germany (33.3%) and the United Kingdom (27.8%) showed relatively high MCP%. In contrast, India (0.0%) and Iran (6.7%) exhibited lower MCP% (Figure 1B; Table 1). It should be noted that MCP% reflects the proportion of multi-country publications within a country’s total output and cannot replace absolute publication counts. For example, China produced 174 publications, which was much higher than Germany’s 18 publications. Therefore, country collaboration results should be interpreted together with total publication output and MCP%. As illustrated in Figure 2A, the United States and China occupied relatively central positions in the international collaboration network. Among the ten most productive institutions, four were based in the United States, two each in Egypt and South Korea, and one each in China and Thailand. Mahidol University (16) ranked first in publication output, followed by Cairo University (17). Harvard University and Northwestern University, both in the United States, were tied for third place with 16 publications each (Figure 2B; Table 2).

**Table 1:** Most relevant countries by corresponding authors of laser treatment for acne scars.

Country	Articles	Articles %	SCP	MCP	MCP %
USA	260	29	226	34	13.1
China	174	19.4	150	24	13.8
Korea	81	9	74	7	8.6
Egypt	52	5.8	48	4	7.7
Thailand	41	4.6	35	6	14.6
Italy	39	4.3	29	10	25.6
Iran	30	3.3	28	2	6.7
India	24	2.7	24	0	0
Germany	18	2	12	6	33.3
United Kingdom	18	2	13	5	27.8
Israel	14	1.6	11	3	21.4
Turkey	14	1.6	14	0	0
Australia	13	1.4	11	2	15.4
Brazil	12	1.3	11	1	8.3
Denmark	11	1.2	11	0	0
Spain	11	1.2	9	2	18.2
Canada	10	1.1	6	4	40
Saudi Arabia	9	1	9	0	0
France	7	0.8	5	2	28.6
Japan	7	0.8	6	1	14.3

MCP, Multiple country publication; SCP, Single country publication.

**Table 2:** Top 10 most relevant affiliations of laser therapy for acne scars.

Affiliation	Articles (*n*)
Mahidol University	30
Cairo University	21
Harvard University	16
Northwestern University	16
Yonsei University	15
Al-Azhar University	14
Laser and Skin Surgery Center of New York	14
Yale University	14
Seoul National University	13
Shanghai Jiao Tong University	13

### 3.2 Journals and co-cited analysis

**Figure 3:**
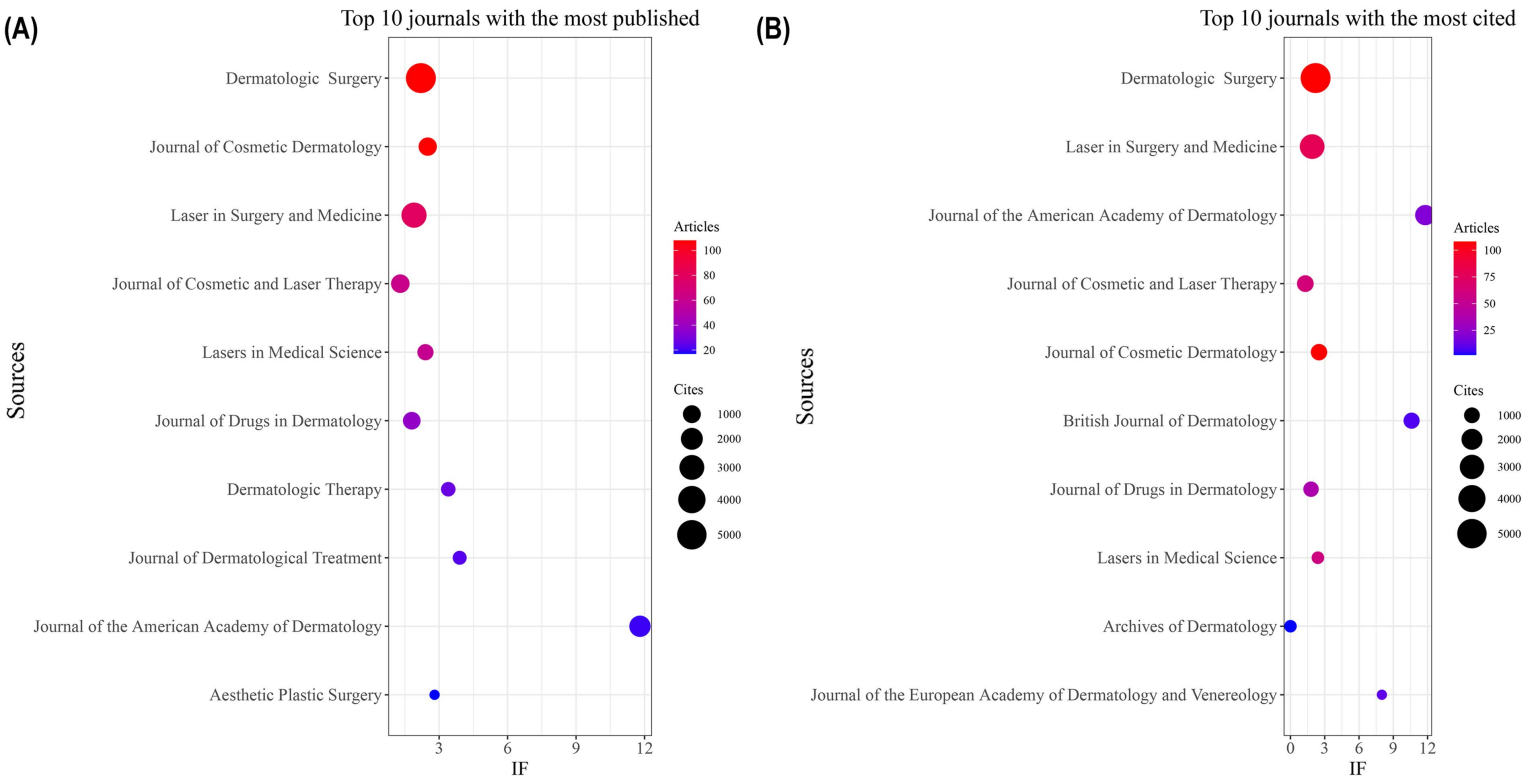
Journals with the largest number of articles published and the journals with the largest number of citations. **(A)** Journals with the largest number of articles published. **(B)** Journals with the largest number of citations.

**Figure 4:**
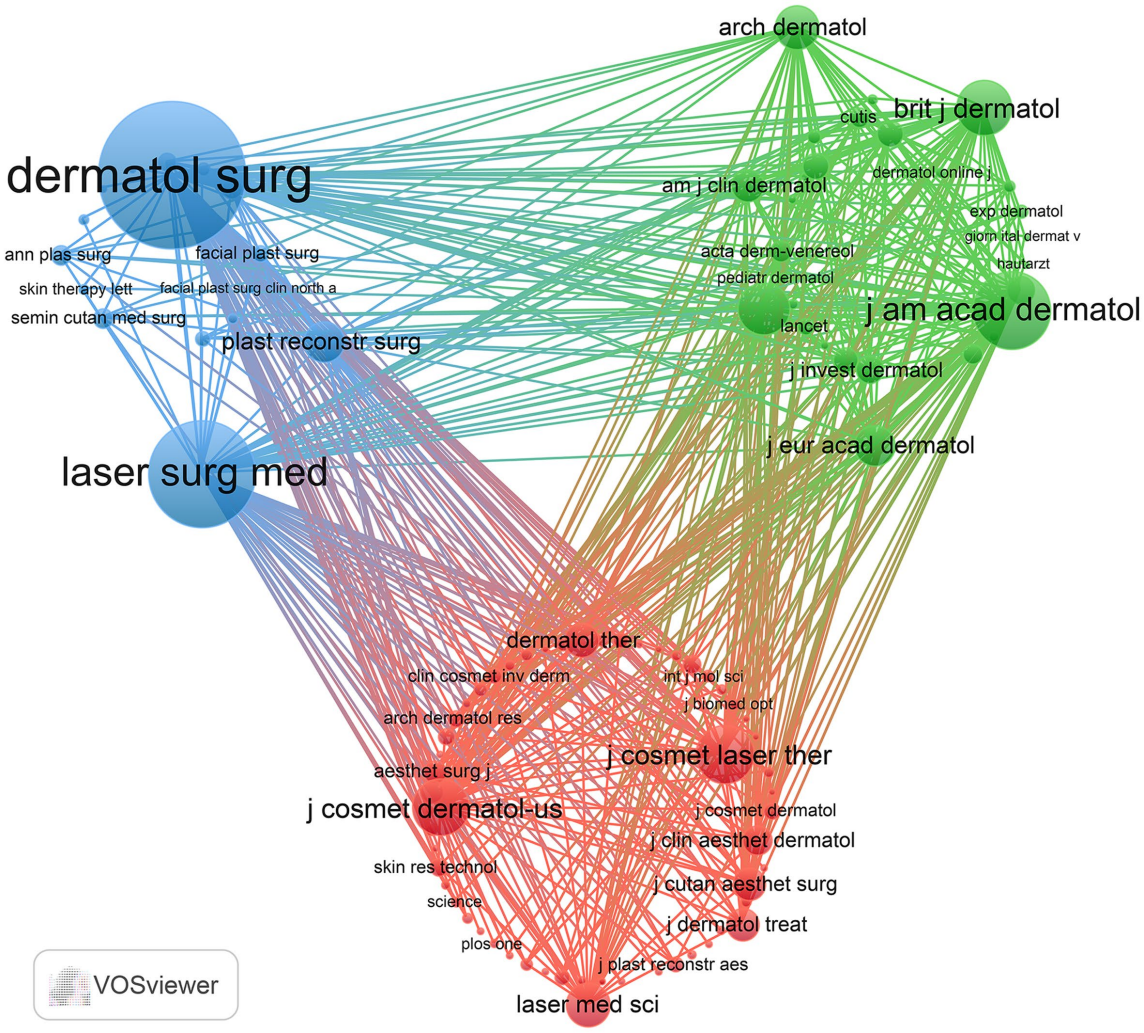
Co-cited journals involved in the research on laser treatment for acne scars.

Prominent journals within this domain were analyzed via R, employing the bibliometrix and ggplot2 packages. In addition, journal co-citation patterns were analyzed with VOSviewer (version 1.6.20.0). The findings showed that the 898 publications were distributed across 133 scholarly journals (Table 3). As shown in Figure 3A, the journals with the highest publication counts were *Dermatologic Surgery* (*n* = 108, IF = 2.2) and *Journal of Cosmetic Dermatology* (*n* = 108, IF = 2.5), followed by *Lasers in Surgery and Medicine* (*n* = 79, IF = 1.9), *Journal of Cosmetic and Laser Therapy* (*n* = 62, IF = 1.3), and *Lasers in Medical Science* (*n* = 59, IF = 2.4). According to Table 4 and Figure 3B, the most frequently cited journals included *Dermatologic Surgery* (*n* = 5,247, IF = 2.2), *Lasers in Surgery and Medicine* (*n* = 3,112, IF = 1.9), *Journal of the American Academy of Dermatology* (*n* = 1832, IF = 11.8), *Journal of Cosmetic and Laser Therapy* (*n* = 1,139, IF = 1.3), and *Journal of Cosmetic Dermatology* (*n* = 1,093, IF = 2.5). Notably, the journal co-citation map showed that *Dermatologic Surgery*, *Lasers in Surgery and Medicine*, and *Journal of the American Academy of Dermatology* were located near the center of the journal co-citation network (Figure 4). Moreover, highly cited journals and high-volume journals overlapped in up to 70% of cases.

**Table 3:** Top 10 journals with the most published articles.

Journal	Documents	Cites	IF(2024)
Dermatologic Surgery	108	5,247	2.2
Journal of Cosmetic Dermatology	108	1,093	2.5
Lasers in Surgery and Medicine	79	3,112	1.9
Journal of Cosmetic and Laser Therapy	62	1,139	1.3
Lasers in Medical Science	59	707	2.4
Journal of Drugs in Dermatology	38	959	1.8
Dermatologic Therapy	27	488	3.4
Journal of Dermatological Treatment	23	429	3.9
Journal of the American Academy of Dermatology	20	1832	11.8
Aesthetic Plastic Surgery	17	254	2.8

**Table 4:** Top 10 journals with the most cited journals.

Journal	Cites	Document	IF (2024)
Dermatologic Surgery	5,247	108	2.2
Lasers in Surgery and Medicine	3,112	79	1.9
Journal of the American Academy of Dermatology	1832	20	11.8
Journal of Cosmetic and Laser Therapy	1,139	62	1.3
Journal of Cosmetic Dermatology	1,093	108	2.5
British Journal of Dermatology	1,049	8	10.6
Journal of Drugs in Dermatology	959	38	1.8
Lasers in Medical Science	707	59	2.4
Archives of Dermatology	704	2	0
Journal of the European Academy of Dermatology and Venereology	643	11	8

### 3.3 Most cited references and reference burst

**Figure 5:**
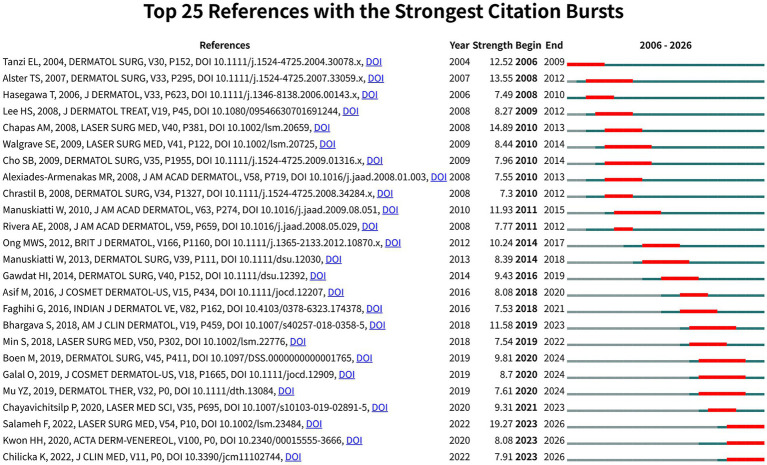
Top 25 references with the strongest citation bursts in the research on laser treatment for acne scars.

The top 20 most locally cited articles in the field of laser therapy for acne scar management were identified using the bibliometrix package in R (Table 5). All selected publications had more than 40 local citations and were distributed across eight distinct journals. Notably, *Dermatologic Surgery* was the most frequently cited journal among the top 20 references, appearing seven times. The three most locally cited articles were: (1) “Successful treatment of acneiform scarring with CO₂ ablative fractional resurfacing”; (2) “The use of fractional laser photothermolysis for the treatment of atrophic scars”; and (3) “Efficacy and safety of a carbon-dioxide ablative fractional resurfacing device for treatment of atrophic acne scars in Asians.” A citation burst analysis was performed using CiteSpace (parameters: top 25; number of emergences = 2; minimum duration: 2). This analysis identified 162 articles exhibiting the most pronounced citation bursts, from which the 25 most significant emerging references were identified (Figure 5). To further identify these burst references, the DOIs of the 25 references shown in Figure 5 were matched with their article titles. The three references with the strongest citation bursts were: (1) “Energy-based devices for the treatment of acne scars: 2021 international consensus recommendations” (strength = 19.27); (2) “Successful treatment of acneiform scarring with CO₂ ablative fractional resurfacing” (strength = 14.89); and (3) “The use of fractional laser photothermolysis for the treatment of atrophic scars” (strength = 13.55). From the perspective of burst period, the three references most closely related to the current research frontiers were: (1) “Energy-based devices for the treatment of acne scars: 2021 international consensus recommendations” (2023–2026); (2) “Combination treatment with human adipose tissue stem cell-derived exosomes and fractional CO₂ laser for acne scars: A 12-week prospective, double-blind, randomized, split-face study” (2023–2026); and (3) “Methods for the improvement of acne scars used in dermatology and cosmetology: A review” (2023–2026).

**Table 5:** Top 20 locally cited references related to laser treatment for acne scars.

Paper	DOI	Local citations
Chapas AM, 2008, Laser Surg Med	10.1002/lsm.20659	100
Alster TS, 2007, Dermatol Surg	10.1111/j.1524-4725.2007.33059.x	99
Manuskiatti W, 2010, J Am Acad Dermatol	10.1016/j.jaad.2009.08.051	99
Goodman GJ, 2006, Dermatol Surg	10.1111/j.1524-4725.2006.32354.x	83
Alexiades-Armenakas MR, 2008, J Am Acad Dermatol	10.1016/j.jaad.2008.01.003	76
Chan NPY, 2010, Laser Surg Med	10.1002/lsm.20974	65
Chan HHL, 2007, Laser Surg Med	10.1002/lsm.20512	64
Geronemus RG, 2006, Laser Surg Med	10.1002/lsm.20310	63
Brauer JA, 2015, Jama Dermatol	10.1001/jamadermatol.2014.3045	63
Cho SB, 2010, J Eur Acad Dermatol	10.1111/j.1468-3083.2009.03551.x	61
Rivera AE, 2008, J Am Acad Dermatol	10.1016/j.jaad.2008.05.029	59
Lee JW, 2011, Dermatol Surg	10.1111/j.1524-4725.2011.01999.x	59
Gawdat HI, 2014, Dermatol Surg	10.1111/dsu.12392	56
Cho SB, 2009, Dermatol Surg-a	10.1111/j.1524-4725.2009.01316.x	51
Ong MWS, 2012, Brit J Dermatol	10.1111/j.1365-2133.2012.10870.x	50
Manuskiatti W, 2013, Dermatol Surg	10.1111/dsu.12030	50
Bhargava S, 2018, Am J Clin Dermatol	10.1007/s40257-018-0358-5	50
Hedelund L, 2012, Laser Surg Med	10.1002/lsm.22048	48
Hasegawa T, 2006, J Dermatol	10.1111/j.1346-8138.2006.00143.x	47
Chrastil B, 2008, Dermatol Surg	10.1111/j.1524-4725.2008.34284.x	45

### 3.4 Keyword clustering and temporal trends

**Figure 6:**
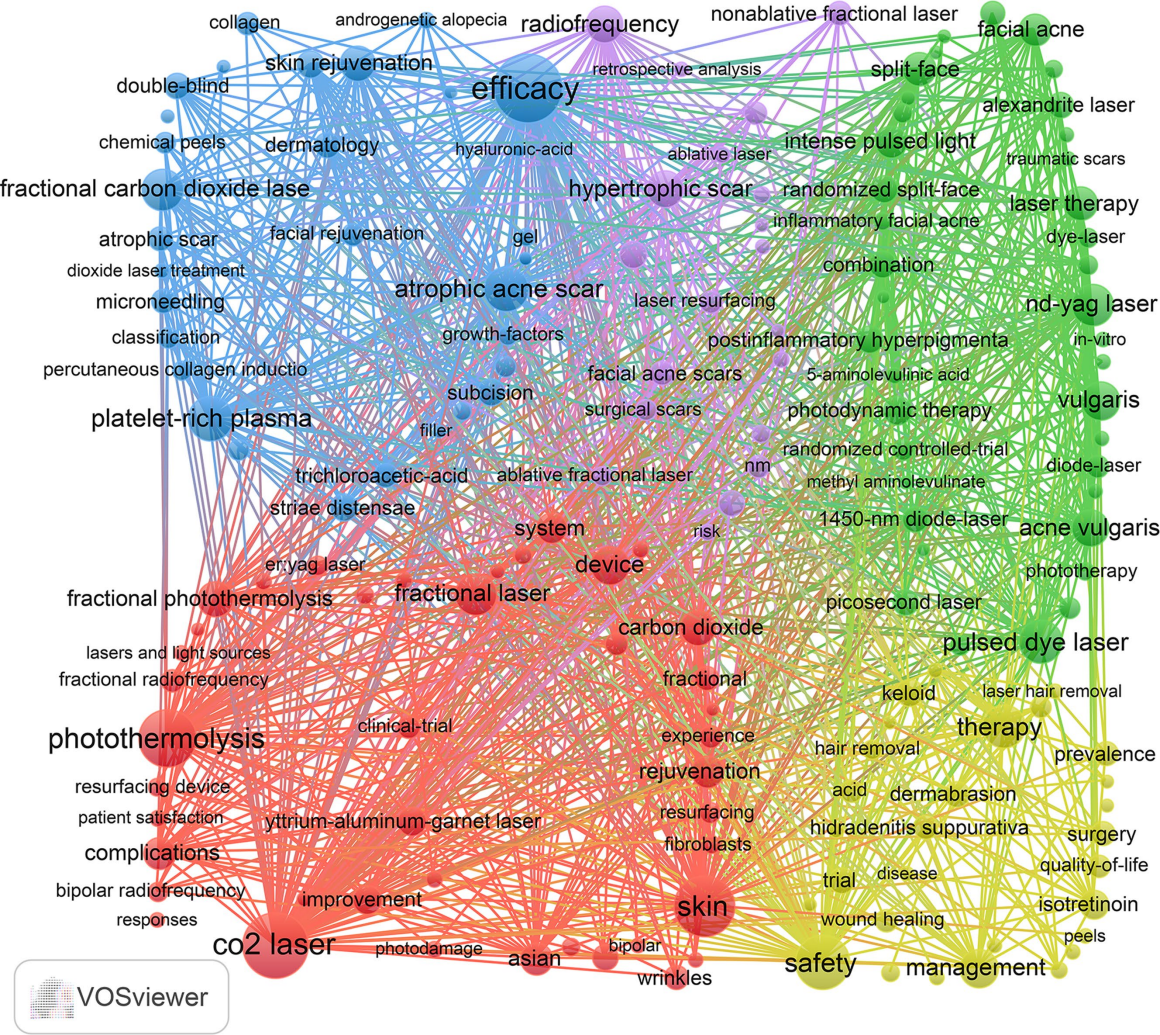
Keyword co-occurrence map of publications on laser treatment for acne scars.

Keyword co-occurrence analysis is an efficient method to explore principal research focuses and developments in a domain. A total of 164 keywords were retrieved using VOSviewer. Table 6 presents the top 20 keywords each appearing more than 50 times highlighting key research priorities. The most commonly used keywords included efficacy (202) followed by CO₂ laser (177) skin (151) photothermolysis (134) and safety (122). Subsequently using a minimum occurrence threshold of seven 164 keywords were selected to construct a keyword co-occurrence map (Figure 6)

**Table 6:** Top 20 keywords related to laser treatment for acne scars.

Rank	Words	Occurrences
1	Efficacy	202
2	CO_2_ laser	177
3	Skin	151
4	Photothermolysis	134
5	Safety	122
6	Atrophic acne scar	84
7	Platelet-rich plasma	82
8	Pulsed dye laser	77
9	Therapy	75
10	Nd-yag laser	72
11	Fractional laser	71
12	Fractional carbon dioxide Laser	64
13	Device	62
14	Vulgaris	61
15	Management	58
16	Hypertrophic scar	57
17	Radiofrequency	56
18	Acne vulgaris	55
19	Carbon dioxide	51
20	Fractional Photothermolysis	50

CO_2_ laser = carbon dioxide laser; nd-yag laser = Neodymium-doped Yttrium Aluminum Garnet laser.

Five distinct clusters, represented by different colors, can be observed in the keyword co-occurrence map. The first cluster (red) includes 42 keywords, such as photothermolysis, fibroblasts, pigmentation, asian skin, and complications. The second cluster (green) contains 39 keywords including 5-aminolevulinic acid, photodynamic therapy, acne vulgaris, inflammatory facial acne, intense pulsed light, diode laser, pulsed dye laser, *propionibacterium acnes*, and randomized controlled trials. The third cluster (blue) comprises 31 keywords, including atrophic acne scars, chemical peels, microneedling, platelet-rich plasma, subcision, filler, and combination therapy. The fourth cluster (yellow) contains 29 keywords such as isotretinoin, guidelines, quality of life, and safety. The fifth cluster (purple) contains 20 keywords, including ablative fractional laser, non-ablative fractional laser, facial acne scars, hypertrophic scars, surgical scars, retrospective analysis, adverse events, and prevention.

**Figure 7:**
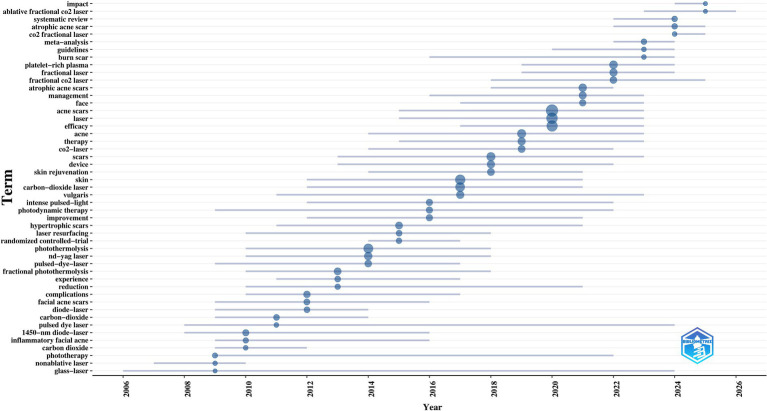
Trend topics in the research on laser treatment for acne scars.

Furthermore, trending topic maps were generated using the bibliometrix package in R (Figure 7). Based on the trend plots shown in Figure 7, the progression and direction of research emphasis within the domain of laser therapy for acne scarring were evaluated. Over the past 20 years, the terms observed in the trend-topic map changed across different periods. Around 2010, the main trend topics included “phototherapy,” “1,450-nm diode-laser,” “pulsed dye laser,” and safety-related terms such as “complications.” During the 2010s, the trend topics were mainly distributed in two aspects. First, terms related to specific laser technologies and treatment strategies were observed, including “fractional photothermolysis,” “co2 laser,” “laser resurfacing,” and “skin rejuvenation.” Second, terms related to more detailed clinical research on acne scars were also observed, including “vulgaris,” “face,” “atrophic acne scars,” “randomized controlled-trial,” and “platelet-rich plasma.” In recent years, trend topics increasingly included terms related to evidence-based medicine, such as “meta-analysis,” “systematic review,” and “guidelines.”

### 3.5 Research hotspots based on WoSCC

Through an analysis of the Web of Science database, we identified the current research hotspots in the field of laser treatment for acne scars. These hotspots can be summarized as follows: (1) Precise laser treatment for different types of acne scars and mechanisms of action; (2) Combined treatment with multiple repair methods using laser as the core technology; and (3) Core challenges and individualized needs in the clinical practice of laser treatment for acne scars.

### 3.6 Clinical evidence directions based on PubMed meta-analyses

This study finally included 25 meta-analyses from the PubMed database. The extracted characteristics and reported evidence from these meta-analyses are summarized in Supplementary material 2. Because the included meta-analyses varied in study types, interventions, outcome indicators, and effect-measure reporting, this section presents a descriptive summary of the main clinical evidence directions rather than interpreting each effect measure individually. Based on intervention types and research questions, these meta-analyses mainly involved three clinical evidence directions: (1) Combination therapy: Designed to overcome the limitations of single-laser treatment in treating acne scars and to achieve synergistic therapeutic effects. (2) Comparative technology assessment: Evaluating the advantages and disadvantages of different laser technologies in acne scar treatment based on evidence-based data. (3) Exploring the effectiveness of integrated traditional Chinese and Western medicine approaches and emerging therapies in the treatment of acne scars.

## 4 Discussion

### 4.1 General information

To better understand research priorities and trends in the field of laser therapy, we conducted a bibliometric analysis and data visualization of 898 papers retrieved from 2006 to March 10, 2026. For annual publication trends, the interpretation was restricted to complete calendar years from 2006 to 2025. Our results showed a steady increase in the total number of publications, with some fluctuations, indicating that this area has sustained research interest and significant potential for development. Among the contributing countries, the United States ranked first in research output (260), followed by China (174) and South Korea (81). Germany and the United Kingdom showed relatively high MCP% among the top ten productive countries, whereas India and Iran showed relatively low MCP%. However, these percentages should be interpreted cautiously together with absolute publication outputs, because countries with smaller publication counts may show larger fluctuations in MCP%. For example, Germany had an MCP% of 33.3% but only 18 publications, whereas China had 174 publications with an MCP% of 13.8%. The United States and China had more connections in the collaboration network and may represent important nodes of international cooperation in this field. Among the ten most productive institutions, four were based in the United States, two each in Egypt and South Korea, and one each in China and Thailand, with Thailand’s Mahidol University leading the way with 30 publications. Notably, the Americas and Asia each account for 40% of the top 10 countries, while Africa accounts for 20%. This distribution indicates that research on the laser treatment of acne scars is particularly prominent in the United States compared to other regions, while Asia and Africa also demonstrate substantial attention to this field. Interestingly, Thailand, despite ranking fifth in total publications, hosts the most productive institution globally. In contrast, although the United States leads in total publications, it has four of the top ten institutions by publication count, with the highest-ranked institution placed third and the others ranked seventh and eighth. This pattern suggests that countries differ in how they allocate scientific research resources and reflects a competitive landscape in the United States. Research on laser therapy for acne scars remains at an early stage, indicating considerable potential for deeper and broader development. Across the 898 published papers, 133 journals were involved. The most prominent journals include *Dermatologic Surgery*, followed by *Lasers in Surgery and Medicine*, *Journal of Cosmetic and Laser Therapy*, as well as *Journal of Cosmetic Dermatology*. Notably, *Dermatologic Surgery* is both the most cited and the most published journal, whereas *Journal of Cosmetic Dermatology*, which ranks equally for publication count, is fifth in terms of citations. Dermatologic Surgery, Lasers in Surgery and Medicine, and Journal of the American Academy of Dermatology were located near the center of the journal co-citation network and may serve as important nodes of knowledge flow in this field. The overlap between highly cited journals and high-output journals may suggest that the dissemination of core knowledge in laser treatment for acne scars is relatively concentrated. This concentration may help maintain academic continuity and research quality; however, it may also indicate a potential risk of academic homogenization or consolidation of research paradigms if publication channels remain overly concentrated. These findings highlight the central role of these journals in dissemination research on laser therapy of acne scars. Current studies suggest that this field holds great promise for future development. Continued research is essential to advance the field, deepen researchers’ insight, and promote the application of novel findings in clinical practice, thereby improving the quality of life for a broader population of patients.

### 4.2 WoSCC-based hot spots and development trends

Based on WoSCC bibliometric results, we analyzed highly cited literature, citation dynamics, keyword co-occurrence, and thematic patterns to identify key research areas within laser-based acne scar treatment. These can be summarized in three key areas: (1) Precise laser treatment for different types of acne scars and mechanisms of action. (2) Combined treatment with multiple repair methods using laser as the core technology. (3) Core challenges and individualized needs in the clinical practice of laser treatment for acne scars.

#### 4.2.1 Precise laser treatment and mechanisms for acne scar subtypes

Early studies primarily focused on evaluating the basic efficacy of various lasers. With technological advances, researchers have shifted their attention to detailed analyses of the mechanisms of action (18). The first cluster (red) was related to the mechanisms of laser treatment for acne scars, tissue responses, and considerations for specific skin types. The second cluster (green) was related to the use of laser or energy-based therapies to suppress active inflammation and intervene in acne scars during the formation stage. Together, these two clusters indicate that research on precise laser treatment has not only focused on tissue remodeling and skin-type-related responses, but also on inflammatory control during the early development of acne scars. It should be noted that the bibliometric results only show the occurrence and clustering of mechanism-related keywords in this field and cannot directly prove specific biological mechanisms or efficacy differences of specific lasers for different scar types. Therefore, the discussion in this section regarding photothermal effects, fibroblasts, collagen remodeling, and applicable scenarios for different lasers is based mainly on keyword signals combined with cautious interpretation of representative studies. For example, representative studies have described that ablative fractional lasers create micro-therapeutic zones through controlled ablation (19). These microscopic thermal injuries may initiate wound-repair processes, including inflammatory cell infiltration, release of related growth factors (20), and remodeling and neogenesis of dermal collagen fibers; therefore, they have been discussed as potentially relevant for deeper acne scars (21). In contrast, non-ablative fractional lasers (e.g., 1,550 nm and 1927 nm) induce controlled dermal thermal damage through gentle and precise photothermal effects without disrupting the epidermis (22). This process has been reported to stimulate fibroblast activity, promote the synthesis of collagen and elastin, and facilitates dermal remodeling, thereby contributing to scar texture improvement (17). Non-ablative lasers have been described as being associated with relatively rapid recovery and minimal side effects (23). Recent studies have employed randomized controlled trials and randomized split-face studies to optimize laser treatment for different types of acne scars (24), which may support the development of more individualized treatment regimens. For instance, clinical studies have reported that pulsed dye laser and long-pulse nd-yag laser may improve the appearance of acne scars and reduce ECCA scores (25). Some studies have reported that ice-pick scars with relatively rich blood supply may respond more favorably to the 585-nm pulsed dye laser (PDL) (26). A possible explanation is based on the principle of selective photothermolysis: hemoglobin within acne scars may preferentially absorb the PDL wavelength, leading to thermal coagulation and microvessel occlusion, thereby potentially reducing inflammation and blood supply. For wider boxcar scars, the 1,064-nm long-pulse nd-yag laser may have potential application value, which may be related to its deeper tissue penetration and absorption by both water and hemoglobin, thereby potentially generating a more uniform thermal effect in the dermis and promoting collagen contraction and remodeling (27, 28). Keyword clustering analyses highlighted terms such as “photolysis,” “light,” “fibroblasts,” “collagen,” and “stem cells,” which may indicate increasing attention to cellular biological responses. The thematic trend map may further reflect a shift from broad investigations of “phototherapy” to more specific technologies such as “fractional photothermolysis” and “co2 laser.” These results may suggest that the field is moving toward more refined and individualized treatment. Future studies may further integrate well-defined cellular and molecular mechanisms, scar morphology, skin color, pathological characteristics, and treatment parameters to optimize laser treatment strategies.

#### 4.2.2 Laser as the core technology in combination with various repair methods

The efficacy of laser therapy alone in treating specific types of acne scars is often limited (29, 30). Analysis of keyword trends and related research demonstrates that treatment strategies have evolved from single-technology approaches to multi-modality combination therapies. The keyword “combination therapy” has emerged as a research focus, reflecting the concept of integrating various biological agents or drugs with laser treatment to achieve synergistic effects. The third cluster (blue) focused on laser-centered combination strategies that integrate other repair techniques for acne scar treatment to achieve synergistic therapeutic effects. Studies have confirmed the benefits of combination therapy. For example, platelet-rich plasma (PRP), which is rich in growth factors, can be combined with fractional lasers to create microchannels. This approach accelerates tissue repair, reduces post-inflammatory erythema, and promotes collagen regeneration (16), thereby significantly improving overall wound healing and treatment efficacy. One study reported the use of oral isotretinoin to control active acne prior to laser therapy (31). Once acne activity is controlled, hyaluronic acid fillers were also reported as a complement to laser therapy (32). The volumizing and supportive effects of hyaluronic acid fillers enhance tissue repair and promote optimal healing after laser treatment (33). Notably, the concept of combination therapy is advancing toward a more “biologically active” and “intelligent” approach (34). For instance, Hyuck Hoon Kwon’s team investigated the application of human adipose stem cell-derived exosomes (ASCE) in combination with fractional CO₂ lasers for treating atrophic acne scars (35). Findings indicated that the combined therapy was significantly more effective than monotherapy. More importantly, exosomes serve both as bioactive agents and engineered carriers (36), reflecting the deep integration of regenerative medicine principles, biofabrication technologies, and energy-based device engineering (37). In the future, combination therapies may achieve a seamless integration of diagnosis and treatment, incorporating real-time imaging, dynamic adjustment of laser parameters, and precise delivery of bioactive carriers. Such advancements will make acne scar treatment increasingly precise, controllable, and personalized (38).

#### 4.2.3 Core challenges and individualized needs in the clinical practice of laser treatment of acne scars

With the widespread adoption of laser therapy for acne scars, the keywords “safety” and “adverse events” have become increasingly prominent in clinical practice (39). The fourth cluster (yellow) focused on the decision-making framework and efficacy evaluation of laser treatment for acne scars. The fifth cluster (purple) was related to the technical spectrum, core indications, and risk management of laser treatment for acne scars. Together, these two clusters may suggest that clinical decision-making, efficacy evaluation, indication selection, and adverse-event prevention are important issues in the clinical practice of laser treatment for acne scars. The citation burst results may suggest that, as increasing attention has been paid to the quality of life of patients with acne scars, laser therapy, as a widely used external intervention, has received broad scholarly attention. Although this approach may show efficacy for certain types of acne scars, the standardization of individualized treatment parameters and the development of safety assurance systems remain insufficient. Therefore, more in-depth and forward-looking studies are needed in this field. There is currently a lack of standardized treatment protocols and long-term safety data to guide practice (40). This gap means that for patients with different types of acne scars and varying skin types, clinicians must often rely on personal experience to set laser parameters, exposing patients to potentially unpredictable risks. For example, Dai et al. demonstrated that treatment of atrophic acne scars in Asian patients using the 2,940-nm ablative fractional erbium laser resulted in mild postinflammatory hyperpigmentation in 30% of patients—a risk significantly higher than that observed with the 1,064-nm picosecond laser (41). In response, the industry has begun exploring more tolerable technologies, such as radiofrequency-based approaches (42), designed to overcome limitations in patient suitability, reduce clinical risk, and provide safer treatment options. Currently, refined and stratified therapeutic strategies have received increasing research attention. Treatment selection considers factors such as the morphology and number of acne scars, Fitzpatrick skin type, and the patient’s acceptable recovery period. Studies have explored multimodal approaches combining chemical exfoliation, fillers, radiofrequency microneedling, laser therapy, and surgical interventions, with the aim of achieving optimal outcomes (43).

In the future, individualized treatment may rely on high-quality, long-term follow-up data and the development of clinical guidelines (44). The integration of artificial intelligence could assist clinicians by automatically analyzing skin images to classify acne scar types and recommend initial laser parameters (45–47). Such algorithms would provide a more objective basis for personalized treatment, minimizing the risk of adverse events such as erythema, postinflammatory hyperpigmentation, infection, or scar aggravation, and better addressing the individual needs of patients (48).

### 4.3 Discussion of clinical evidence directions based on PubMed meta-analyses

Based on a descriptive summary of the PubMed-indexed meta-analyses, the reported clinical evidence directions in the treatment of acne scars were grouped into three main areas: (1) Combination therapy, which aims to overcome the limitations of single-laser treatment for acne scars and achieve synergistic therapeutic effects. (2) Technical comparison: The advantages and disadvantages of different laser techniques for treating acne scars are evaluated based on evidence-based findings. (3) Exploring the effectiveness of integrated traditional Chinese and Western medicine approaches and emerging therapies in the treatment of acne scars.

#### 4.3.1 Combination therapies to achieve synergy

Current research has shifted from merely evaluating the efficacy of lasers to further exploring their therapeutic potential. Combination therapy has emerged as a central focus, reflecting the clinical goal of improving treatment outcomes and patient quality of life. Two meta-analyses, each incorporating multiple randomized controlled trials (RCTs), compared laser therapy combined with platelet-rich plasma (PRP) to laser therapy alone (49, 50). Original meta-analyses suggested that PRP combined with laser therapy may have potential advantages over laser therapy alone in scar improvement, patient satisfaction, and recovery time, with detailed effect measures and corresponding source meta-analyses provided in Supplementary material 2. Similarly, the combination of hyaluronic acid with laser therapy has gained increasing attention (51). The original meta-analysis suggested that this approach may reduce ECCA scar scores, shorten the duration of scab formation and shedding, improve patient satisfaction, and reduce the risk of post-treatment hyperpigmentation, with detailed effect measures and corresponding source meta-analyses provided in Supplementary material 2. Overall, laser combined with bioactive materials or repair dressings may help overcome the limitations of laser monotherapy and represents a synergistic strategy worthy of attention in acne scar treatment.

#### 4.3.2 Comparison of different treatment techniques for acne scars

There is an increasing emphasis on the individualized selection of laser technologies in the management of acne scars. Meta-analyses compare key indicators such as scar improvement, pain, recovery time, and risk of pigmentation across different laser types and specific scar presentations. For example, a 2025 meta-analysis by Ke R et al. compared the efficacy and safety of non-ablative lasers and ablative lasers for acne scarring (52). The original meta-analysis reported that the advantages of the two laser types varied across outcome indicators: non-ablative lasers showed advantages in safety and recovery-related outcomes, such as pain and erythema duration, whereas ablative lasers showed better scar improvement in observer-based and patient-driven evaluations, with detailed effect measures and corresponding source meta-analyses provided in Supplementary material 2. Therefore, these findings suggest that efficacy, pain, recovery time, and patient tolerance may be relevant considerations when comparing different laser types. Regarding the treatment of atrophic scars, a 2024 meta-analysis systematically evaluated the advantages of CO₂ lasers versus Er: YAG lasers (53). The original meta-analysis reported that CO₂ lasers had a significantly higher effective rate and a shorter recovery period than Er: YAG lasers. However, CO₂ lasers were associated with greater intraoperative pain and a longer duration of postoperative erythema, with detailed effect measures and corresponding source meta-analyses provided in Supplementary material 2. These findings underscore the need for high-level evidence beyond individual experience. Meta-analyses may provide a reference when considering treatment techniques for different types of acne scars.

#### 4.3.3 New exploration of acne scar treatment technologies

Exploring novel treatments for acne scars remains a key focus in current clinical practice. By descriptively summarizing the findings reported in the included PubMed Meta-Analyses, this section aims to describe the emerging treatment strategies examined in acne scar management. A 2025 meta-analysis reported that the combination of fractional CO₂ laser and acupuncture—enhancing anti-inflammatory and analgesic effects—significantly reduced the ECCA score, improved the overall response rate, and enhanced overall treatment efficacy without increasing risk, with detailed effect measures and corresponding source meta-analyses provided in Supplementary material 2 (54). The growing interest in novel therapies underscores the diversity of advancements in the field and highlights the trend toward innovative, integrative, and patient-centered approaches in acne scar management.

In summary, current studies on laser-based therapies for acne scars primarily focus on optimizing technologies to enable precise, personalized, and effective treatment strategies.

### 4.4 Limitations

This study has several limitations. First, we included only the Web of Science Core Collection (WoSCC) database for bibliometric analysis, which may have excluded valuable literature from other databases. Because this study was based only on WoSCC data for bibliometric analysis, country rankings, institution distribution, and journal influence may be affected by WoSCC coverage. English-language journals, international journals, and related countries and institutions more fully indexed by WoSCC may be relatively overrepresented, whereas regional or non-WoSCC-indexed studies may be underestimated. Because no cross-database sensitivity analysis was performed, the magnitude of this potential bias could not be quantitatively assessed. Therefore, the relevant bibliometric results should be interpreted as descriptive findings based on WoSCC data rather than as a complete representation of all publications in this field. Second, PubMed data were used only to extract Meta-Analysis articles as supplementary descriptive evidence regarding the main reported clinical evidence directions. PubMed records were not merged with the WoSCC dataset and were not included in the WoSCC-based keyword co-occurrence, co-citation, or trend mapping analyses. Therefore, the keyword and trend results of this study mainly reflect the bibliometric characteristics of WoSCC and cannot fully represent the overall distribution of all related clinical studies indexed in PubMed. Third, the search strategy relied primarily on the terms “Acne Scar*” and “Laser*” in WoSCC and the corresponding Title/Abstract terms in PubMed. Although this focused strategy improved the reproducibility of the retrieval process, it may have missed relevant studies indexed under alternative terminology or specific device names without explicitly using these terms. Therefore, the findings should be interpreted within the scope of the predefined search strategy. Fourth, country productivity analysis used raw publication counts and was not normalized by population size, gross domestic product (GDP), or research expenditure. Therefore, comparisons of publication output across countries may be subject to bias and should be interpreted cautiously. Fifth, the WoSCC dataset was limited to Articles and Reviews to focus on more mature research findings. This approach may have excluded other types of publications, potentially overlooking emerging technologies or early-stage clinical case discussions, resulting in a narrower perspective. Because reviews usually have citation patterns that differ from those of original research articles, the combined analysis of Articles and Reviews in this study may have influenced the analyses of highly cited publications and journal influence. These citation-related findings should therefore be regarded as descriptive presentations of overall knowledge dissemination rather than as rankings of the influence of original research alone. Finally, our search was limited to English-language literature, possibly excluding key studies in languages other than English. Despite these limitations, this study provides reliable and valuable insights. It offers a structured characterization of the field, enabling researchers to deepen their understanding of its overall development and identify potential directions for future investigation.

## 5 Conclusion

This study mapped the major research hotspots concerning laser interventions for acne scarring and drew the following key conclusions:

1. Laser therapy for acne scars attracts considerable interest worldwide. The United States, as a leading contributor, has made important advancements in this field, alongside several countries in Asia and Africa. Moreover, based on the combined interpretation of MCP% and total publication output, Germany and the United Kingdom showed relatively high proportions of multi-country publications among the top ten productive countries, although this did not necessarily imply a greater absolute volume of international collaboration.
2. *Dermatologic Surgery* is the most prominent journal in this field, particularly regarding the number of publications and citations. *Journal of Cosmetic Dermatology*, *Lasers in Surgery and Medicine*, and *Journal of Cosmetic and Laser Therapy* also exert considerable influence.
3. Studies of laser therapy for acne scars have increasingly focused on elucidating precise mechanisms based on the principles of selective photothermolysis.
4. Laser-based combination treatment strategies have emerged as a prominent trend in acne scar research.
5. Researchers in the field of laser treatment for acne scars have increasingly focused on risk-related challenges and individualized patient needs.
6. Overall, this study offers important perspectives on future research directions in laser-based acne scar therapy by examining the development trajectory, knowledge structure, and emerging trends in the field. These results enable investigators to understand the current state of research, while highlighting potential avenues for future investigation. Based on WoSCC keyword clustering results and the descriptive findings reported in PubMed Meta-Analyses, differences in acne scar types, skin color, and individual patient characteristics have received increasing research attention. Further investigation of individualized laser treatment parameters and combination protocols may represent an important future research direction. However, this study did not establish specific treatment parameters or stratified treatment models, and these directions still require further validation through high-quality clinical studies. Furthermore, these findings may provide a reference for the future development of the field from simple technical improvements toward more standardized, evidence-based, and precise approaches.

## Data Availability

The original contributions presented in the study are included in the article/Supplementary material, further inquiries can be directed to the corresponding author.

## Supplementary material

SUPPLEMENTARY FIGURE S1 Flow diagram of the literature retrieval and screening process for WoSCC records and PubMed meta-analyses.

SUPPLEMENTARY MATERIAL 1 Consolidate Keyword List Following Synonym Merging.

SUPPLEMENTARY MATERIAL 2 Included meta -analyses on photoelectric treatments for acne scars and their key findings.
