# Supplementary material for: Laser treatment of acne scars: a bibliometric analysis and descriptive summary of clinical evidence directions over the past 20 years
Source: Front Med (Lausanne). 2026 Jul 22;13:1875002. doi: 10.3389/fmed.2026.1875002 (PMC13440076; doi:10.3389/fmed.2026.1875002)
Supplement: Supplementary file 1 [file Supplementary_file_1.docx]

|  | Label | Replace by |
| --- | --- | --- |
| 1 | acne scarring | acne scar |
| 2 | acne scars | acne scar |
| 3 | acne-vulgaris | acne vulgaris |
| 4 | asian patients | asian |
| 5 | asians | asian |
| 6 | atrophic scars | atrophic scar |
| 7 | atrophic acne scars | atrophic acne scar |
| 8 | burn scars | burn scar |
| 9 | carbon-dioxide | carbon dioxide |
| 10 | co2 | carbon dioxide |
| 11 | co2-laser | co2 laser |
| 12 | carbon-dioxide laser | co2 laser |
| 13 | er-yag laser | er:yag laser |
| 14 | fractional lasers | fractional laser |
| 15 | fractional co2 laser | fractional carbon dioxide laser |
| 16 | fractional co2-laser | fractional carbon dioxide laser |
| 17 | hypertrophic scars | hypertrophic scar |
| 18 | intense pulsed-light | intense pulsed light |
| 19 | keloids | keloid |
| 20 | lasers | laser |
| 21 | laser treatment | laser therapy |
| 22 | non-ablative fractional laser | nonablative fractional laser |
| 23 | post-inflammatory hyperpigmentation | postinflammatory hyperpigmentation |
| 24 | pulsed dye-laser | pulsed dye laser |
| 25 | pulsed-dye-laser | pulsed dye laser |
| 26 | scarring | scar |
| 27 | scars | scar |
| 28 | striae-distensae | striae distensae |
| 29 | treatment | therapy |
| 30 | yag | yttrium-aluminum-garnet |
| 31 | prp | platelet-rich plasma |
| 32 | acne inversa | hidradenitis suppurativa |
| 33 | rhytides | wrinkles |
| 34 | YAG laser | yttrium-aluminum-garnet laser |
| 35 | co2 fractional laser | fractional carbon dioxide laser |

Note: “Label” refers to the original keyword extracted from the WoSCC records before standardization. “Replaced by” refers to the standardized keyword used in the final keyword co-occurrence analysis after merging synonymous terms, spelling variants, abbreviations, and singular/plural forms.
